# Supplementary material for: Implementation of colorectal cancer screening interventions in low-income and middle-income countries: a scoping review protocol
Source: BMJ Open. 2020 Jun 11;10(6):e037520. doi: 10.1136/bmjopen-2020-037520 (PMC7295404; doi:10.1136/bmjopen-2020-037520)
Supplement: Supplementary data [file bmjopen-2020-037520supp001.pdf]

Supplementary materials 1: Definition of income groupings

Definition of income groupings has been sourced from the World Bank and is based on gross national income (GNI): https://datahelpdesk.worldbank.org/knowledgebase/articles/906519-world-bank-country-and-lending-groups

Low-income: Low-income economies are defined as those with a GNI per capita, calculated using the World Bank Atlas method, of $1,025 or less in 2018.

Lower-middle income: Lower-middle income economies are defined as those with a GNI per capita, calculated using the World Bank Atlas method, between $1,026 and $3,995.

Upper-middle income: Upper-middle income economies are defined as those with a GNI per capita, calculated using the World Bank Atlas method, between $3,996 and $12,375.

High-income: High-income economies are defined as those with a GNI per capita, calculated using the World Bank Atlas method, of $12,376 or more.

Schliemann D, *et al. BMJ Open* 2020; 10:e037520. doi: 10.1136/bmjopen-2020-037520

Supplementary materials 2: Search terms and strategy as devised for MEDLINE

1 exp Colorectal Neoplasms/

2 bowel cancer.mp.

3 early diagnosis/ OR "early detection of cancer"/

4 Mass Screening/

5 (f$cal occult blood test OR fobt OR f$cal immunochemical test OR fit OR colonoscopy OR sigmoidoscopy).mp. [mp=title, abstract, original title, name of substance word, subject heading word, floating sub-heading word, keyword heading word, organism supplementary concept word, protocol supplementary concept word, rare disease supplementary concept word, unique identifier, synonyms]

6 Developing Countries/

7 (Angola* OR India* OR Papua New Guinea OR Bangladesh* OR Indonesia* OR Philippin* OR Bhutan OR Kenya* OR Sao Tom* OR Bolivia* OR Kiribati OR Senegal* OR Cabo Verde* OR Kyrgyz* OR Solomon Island* OR Cambodi* OR Lao* OR Sudan* OR Cameroon* OR Lesotho OR Timor Leste* OR Comoros OR Mauritania* OR Tunisia* OR Congo OR Micronesia OR Ukrain* OR Cote d'Ivoire OR Moldov* OR Uzbekistan* OR Djibouti OR Mongolia* OR Vanuatu Egypt* OR Morocc* OR Vietnam* OR El Salvador OR Myanmar* OR Gaza* OR Eswatini OR Nicaragua* OR Zambia* OR Ghana* OR Nigeria* OR Zimbabwe* OR Honduras* OR Pakistan* OR Argentin* OR Paraguay* OR Mexic* OR Venezuel* OR Costa Rica* OR Cuba* OR Jamaica* OR Guatemala* OR Peru* OR Dominican Republic* OR Ecuador* OR Colombia* OR Brazil* OR Chin* OR Sri Lanka* OR Malay* OR Thai* OR Turk* OR Iran* OR Albania* OR South Africa* OR Maurit* OR Namib* OR Algeria* OR Botswana* OR Bulgaria* OR Fiji* OR Gabon* OR Nauru* OR American Samoa* OR Georgia* OR North Macedonia* OR Grenada* OR Armenia* OR Azerbaijan* OR Guyana* OR Romania* OR Belarus* OR Russia* OR Belize* OR Iraq* OR Samoa* OR Bosnia* OR Herzegovina* OR Serbia* OR Jordan* OR Kazakhstan* OR Kosovo* OR St Lucia* OR Lebanon* OR Grenadin* OR Suriname OR Maldives OR Tonga* OR Dominica OR Marshall Islands OR Turkmenistan OR Equatorial Guinea OR Tuvalu OR Montenegro* OR Liby* OR Afghan* OR Guinea* OR Sierra Leone OR Benin* OR Haiti* OR Somalia* OR Burkina Faso* OR Korea OR South Sudan* OR Burundi* OR Liberia* OR Syria* OR Central African Republic OR Madagascar* OR Tajikistan* OR Chad* OR Malawi* OR Tanzania* OR Congo* OR Mali* OR Togo* OR Eritrea* OR Mozambique* OR Uganda* OR Ethiopia* OR Nepal OR Yemen* OR Gambia* OR Guinea* OR Rwanda*).mp. [mp=title, abstract, original title, name of substance word, subject heading word, floating sub-heading word, keyword heading word, organism supplementary concept word, protocol supplementary concept word, rare disease supplementary concept word, unique identifier, synonyms]

Schliemann D, *et al. BMJ Open* 2020; 10:e037520. doi: 10.1136/bmjopen-2020-037520

8	1 OR 2
9	3 OR 4 OR 5
10	6 OR 7
11	8 and 9 and 10
12	limit 11 to (English language and humans)

Schliemann D, *et al. BMJ Open* 2020; 10:e037520. doi: 10.1136/bmjopen-2020-037520
