# Supplementary material for: Implementation of colorectal cancer screening interventions in low-income and middle-income countries: a scoping review protocol
Source: BMJ Open. 2020 Jun 11;10(6):e037520. doi: 10.1136/bmjopen-2020-037520 (PMC7295404; doi:10.1136/bmjopen-2020-037520)
Supplement: Author's manuscript [file bmjopen-2020-037520.draft_revisions.pdf]

BMJ Open is committed to open peer review. As part of this commitment we make the peer review history of every article we publish publicly available.

When an article is published we post the peer reviewers' comments and the authors' responses online. We also post the versions of the paper that were used during peer review. These are the versions that the peer review comments apply to.

The versions of the paper that follow are the versions that were submitted during the peer review process. They are not the versions of record or the final published versions. They should not be cited or distributed as the published version of this manuscript.

BMJ Open is an open access journal and the full, final, typeset and author-corrected version of record of the manuscript is available on our site with no access controls, subscription charges or pay-per-view fees (http://bmjopen.bmj.com).

If you have any questions on BMJ Open's open peer review process please email info.bmjopen@bmj.com

BMJ Open

Implementation of colorectal cancer screening interventions in low- and middle-income countries: a scoping review protocol

Journal:	*BMJ Open*
Manuscript ID	bmjopen-2020-037520
Article Type:	Protocol
Date Submitted by the Author:	05-Feb-2020
Complete List of Authors:	Schliemann, Desiree ; Queen's University Belfast, Centre for Public Health<br>Ramanathan, Kogila; Monash University - Malaysia Campus<br>O'Neill, Ciaran; Queen's University Belfast, Centre for Public Health<br>Kee, Frank; Queen's University Belfast, Centre for Public Health<br>Su, Tin ; Monash University - Malaysia Campus<br>Donnelly, Michael; Queen's University Belfast, Centre for Public Health
Keywords:	Adult oncology < ONCOLOGY, PUBLIC HEALTH, International health services < HEALTH SERVICES ADMINISTRATION & MANAGEMENT

SCHOLARONE™
Manuscripts

I, the Submitting Author has the right to grant and does grant on behalf of all authors of the Work (as defined in the below author licence), an exclusive licence and/or a non-exclusive licence for contributions from authors who are: i) UK Crown employees; ii) where BMJ has agreed a CC-BY licence shall apply, and/or iii) in accordance with the terms applicable for US Federal Government officers or employees acting as part of their official duties; on a worldwide, perpetual, irrevocable, royalty-free basis to BMJ Publishing Group Ltd ("BMJ") its licensees and where the relevant Journal is co-owned by BMJ to the co-owners of the Journal, to publish the Work in this journal and any other BMJ products and to exploit all rights, as set out in our licence.

The Submitting Author accepts and understands that any supply made under these terms is made by BMJ to the Submitting Author unless you are acting as an employee on behalf of your employer or a postgraduate student of an affiliated institution which is paying any applicable article publishing charge ("APC") for Open Access articles. Where the Submitting Author wishes to make the Work available on an Open Access basis (and intends to pay the relevant APC), the terms of reuse of such Open Access shall be governed by a Creative Commons licence – details of these licences and which Creative Commons licence will apply to this Work are set out in our licence referred to above.

Other than as permitted in any relevant BMJ Author's Self Archiving Policies, I confirm this Work has not been accepted for publication elsewhere, is not being considered for publication elsewhere and does not duplicate material already published. I confirm all authors consent to publication of this Work and authorise the granting of this licence.

For peer review only

Title: Implementation of colorectal cancer screening interventions in low- and middle-income countries: a scoping review protocol

Authors: Désirée Schliemann[1], Kogila Ramanathan[2], Ciaran O'Neill[2], Frank Kee[2], Tin Tin Su[2], Michael Donnelly[1]

[1] Centre for Public Health and UKCRC Centre of Excellence for Public Health, Queen's University Belfast, Belfast, UK

[2] South East Asia Community Observatory (SEACO), Jeffrey Cheah School of Medicine and Health Sciences, Monash University Malaysia

Corresponding author: Désirée Schliemann/ d.schliemann@qub.ac.uk

For peer review only

For peer review only - http://bmjopen.bmj.com/site/about/guidelines.xhtml

Abstract

Introduction: Colorectal cancer (CRC) imposes a significant global burden of disease. CRC survival rates are much lower in low- and middle-income countries (LMICs). Screening tends to lead to an improvement in cancer detection and the uptake of available treatments and, in turn, to better chances of cancer survival. Most evidence on CRC screening interventions comes from high-income countries and there is a need to map the available literature on CRC screening interventions and their uptake in LMIC countries.

Methods and analysis: We will conduct a scoping review according to the framework proposed by Arksey and O'Malley (2005). We will search MEDLINE, EMBASE, Web of Science and Google Scholar using a combination of terms such as 'colorectal cancer', 'screening' and 'low-middle-income countries'. Studies of CRC screening interventions/programmes conducted in the general adult population in LMICs, published in the English language before January 2020 will be included in this review. The title and abstract screen will be conducted by one reviewer and two reviewers will screen full-texts and extract data from included papers, independently, into a data charting template that will include criteria from an adapted TIDieR checklist and implementation considerations. The presentation of the scoping review will be reported according to PRISMA-ScR guidance.

Ethics and dissemination: There are no ethical concerns. The results will be used to inform colorectal screening interventions in low-middle-income countries. We will publish the findings in a peer-reviewed journal and present them at relevant conferences.

Key words: colorectal cancer, bowel cancer, screening, faecal occult blood test, faecal immunochemical test, low-middle income countries

For peer review only - http://bmjopen.bmj.com/site/about/guidelines.xhtml

Strengths and limitations of this study

- This scoping review will map the literature on colorectal cancer screening in low- and middle-income countries, thus highlighting opportunities and challenges for those countries and informing future global health research.
- The approach presented here will provide a timely synthesis of the literature in this field.
- The inclusion criteria are broad in order to 'paint' a comprehensive picture.
- We will include only studies published in the English language.
- The review uses the World Bank classification rather than the Human Development Index to define middle-income countries - some countries that the HDI define as high-income countries may be classed as upper-middle income by the World Bank.

For peer review only

For peer review only - http://bmjopen.bmj.com/site/about/guidelines.xhtml

Introduction

Colorectal cancer (CRC) is the third most common cancer worldwide with an age-standardised incidence rate of 19.7/100,000 for males and females combined and it remains the second most common cause of cancer deaths globally (9.2%) [1]. Large disparities exist in CRC incidence and mortality rates between high- and low-income countries. In Oceania, Europe and North America, CRC mortality rates are about one-third that of incidence rates, whereas in Asia, Latin America and the Caribbean mortality rates are half that of incidence rates, and in Africa mortality rates are two-thirds that of incidence rates [1]. Arnold et al also demonstrated that CRC incidence and mortality correlate with the human development index (HDI), i.e. low- and middle-income countries (LMICs) have experienced a rapid increase in CRC incidence and mortality whereas rates in high-income countries are currently stabilising or decreasing [2]. Rapid yearly increases in CRC incidence of up to 2% have been recorded for some middle-income countries such as Brazil and Costa Rica since the early 2000s [3] and in mortality rates in the Philippines (5.7%) and Belarus (3.7%) over a 10-year period [2]. It has also been estimated that CRC incidence in Africa is expected to increase by 85% by 2030 [4]. Arguably, aging populations and lifestyle changes that more commonly resemble Western lifestyles in LMIC are the main reasons for the increasing trends as well as advancements in cancer detection [2].

The International Agency for Cancer Research (IARC) suggest that there is sufficient evidence in relation to reduced CRC mortality to recommend screening (biannually) for CRC [5]. Screening is most effective when it is delivered in a way that detects cancer at an early stage, i.e. stage 1 or 2 [6], when CRC patients are often asymptomatic. The guaiac faecal occult blood test (gFOBT) and the faecal immunochemical test for haemoglobin (FIT) are non-invasive, inexpensive stool tests that screen for small amounts of blood and that tend to be offered to asymptomatic, ‘at-risk’ individuals (e.g. people at a certain age or with a CRC family history). Invasive, visual screening techniques such as colonoscopy and flexible sigmoidoscopy are offered, generally, to symptomatic and high-risk individuals or those with a positive gFOBT/ FIT. In the last ten years, many high-income countries have introduced organised, population-based cancer screening programmes and follow a systematic approach whereby anyone over a certain age (e.g. 50 years) is invited to complete a stool test at regular (e.g. two-

For peer review only - http://bmjopen.bmj.com/site/about/guidelines.xhtml

yearly) intervals. Screening interventions are recommended only if high quality treatment and follow-up can be delivered [5] and, therefore, the readiness of a country's health system to implement a screening programme is crucial [7]. Some middle-income countries offer opportunistic screening, (e.g. sporadic stool tests to individuals at-risk who attend a clinic) but lack financial resources and the infrastructure to support organised screening (i.e. qualified staff, screening centres, and cancer registries). Low income countries appear to have no or limited CRC screening or treatment facilities in place [8]. Many LMICs also lack cancer registries and therefore recording of cases and cancer control efforts are a major challenge [9–11]. Thus, evaluation reports of CRC screening interventions come mainly from high-income countries and most CRC interventions and programmes were delivered at least partly through post, i.e. invitation letters to attend screening and/or mailed stool-test kits to be completed at home [12,13]. The discrepancies between CRC incidence globally and the availability of opportunistic or population-based screening remain a concern [8] and the rising CRC incidence and high mortality rates in LMICs countries require efforts to improve CRC outcomes.

Review objectives

The purpose of this scoping review is to review the global health literature, map evidence from peer-reviewed and grey literature, examine programmes and interventions that aim to improve CRC screening uptake in LMICs countries and highlight research gaps and opportunities. Findings will be used to guide future CRC screening efforts in countries with limited resources. We will use the classification by the World Bank to define countries in terms of lower-middle income, upper-middle income and low-income economies (Supplementary materials 1).

Methods and analysis

We will follow the research approach that conforms with the Preferred Reporting Items for Systematic Reviews and Meta-Analysis extension for Scoping Reviews (PRISMA-ScR) [14]. A scoping review is the most appropriate type of review given the underexplored nature of this area and the need for

For peer review only - http://bmjopen.bmj.com/site/about/guidelines.xhtml

iterative-style searching of multiple sources. The scoping review will be guided by the methodological five-step framework developed by Arksey and O'Malley [15] as outlined below.

Stage 1: identifying the research question

The review will aim to scope reports in the public domain regarding the content delivered, implementation, uptake and effectiveness of CRC screening interventions in low- and middle-income countries. In particular, the review will attempt to answer the following research questions:

1) What intervention/programme modalities have been implemented to encourage people to attend CRC screening?
2) What are the opportunities and challenges in terms of implementing CRC screening interventions/ programmes?
3) What are the findings (qualitative and quantitative) from those interventions/programmes in terms of, for example, implementation, reach, uptake, engagement (including differences between rural and urban areas) and costs/resources?

Stage 2: identifying relevant studies

We will search MEDLINE, EMBASE, Web of Science, and Google Scholar for relevant literature. Furthermore, we will hand search the reference lists of relevant reviews and studies and Google Web for unpublished reports and briefings. In addition, we will contact experts in the field of CRC screening in LMIC to identify additional programmes that may not have been published in the scientific literature. We have devised an initial search strategy (with the expertise of an experienced subject librarian) for MEDLINE (Supplementary material 2) which will be adapted for other databases. Search terms revolve around the three categories related to 'colorectal cancer', 'screening' and 'LMIC countries.

Stage 3: study selection

For peer review only - http://bmjopen.bmj.com/site/about/guidelines.xhtml

The lead author (DS) will conduct the title and abstract screen and two authors (DS and KR) will screen the retrieved full-texts for inclusion in this scoping review based on broad inclusion and exclusion criteria. Inconsistencies between reviewers will be discussed with a third reviewer (MD or TTS). Our review will focus on dedicated CRC screening interventions or programmes and will include any intervention explicitly designed to encourage the public to attend CRC screening and was published before January 2020. National screening programmes as well as screening programmes delivered at lower population aggregates and programmes of any duration will be included. Only studies conducted in LMICs targeting the general adult population (18 years or older) or populations 'at-risk' for CRC will be considered for inclusion. Studies targeting patients and health care professionals will be excluded from this review. We will only include studies published in the English language and will not apply restrictions concerning the year of publication.

Stage 4: charting the data

We will chart the data from the included studies according to categories in an adapted TIDieR checklist for the reporting of interventions [16] in order to illuminate key components and facilitate the production of standardised descriptions. The following fields will be added to the domains of the TIDieR checklist and charted accordingly: 'reference information', 'study design' and 'for whom' (i.e. target population) (Table 1). Additionally, we will chart data on implementation considerations to identify key concepts that influence dissemination and successful implementation of evidence-based programmes. This will be guided by a template developed by Tierney et al [17] which includes ten factors (Table 2), one of which overlaps with the TIDieR checklist (i.e. intervention 'fidelity') and which therefore will only be addressed under implementation considerations. Both checklists have been piloted and deemed appropriate by the team. Two authors (DS and KR) will independently extract information and any discrepancies will be resolved in discussion with a third author (MD or TTS)

Stage 5: collating, summarising and reporting the results

For peer review only - http://bmjopen.bmj.com/site/about/guidelines.xhtml

We will summarise the data extracted from each study and present the findings in a series of detailed tables that will be organised according the TIDieR and implementation science domains, and marshalled to the content of each review question (e.g. intervention) and then grouped or aggregated according to country, region and income level. In particular, the review will present the barriers and enablers to the successful implementation and uptake of CRC screening in LMICs and resource-constrained settings.

Patient and public involvement

The results of the protocol will be shared and discussed with key stakeholders in various global health studies, particularly in relation to our studies in rural communities in Malaysia.

Ethics and dissemination

This paper presents the protocol for a scoping review of CRC screening interventions in low- and middle-income countries. Ethics approval is not necessary as the data will be collected from publically available resources. This review will advance the knowledge on CRC screening programmes for researchers, general practitioners, public health practitioners and policy makers working in low- and middle-income countries. The results will be disseminated through a peer-reviewed journal and will be presented at relevant conferences.

For peer review only - http://bmjopen.bmj.com/site/about/guidelines.xhtml

Contributors

All authors contributed to the development of the research question and objectives. DS drafted the manuscript and MD led the editing of the manuscript. All authors read and approved the final version of the manuscript.

Funding

This research is funded by the Medical Research Council (UK) Global Challenges Research Fund.

Competing interests

None declared.

Patient consent for publication

Not required.

For peer review only

For peer review only - http://bmjopen.bmj.com/site/about/guidelines.xhtml

References

1 International Agency for Research on Cancer. GLOBOCAN. 2018. http://gco.iarc.fr/today/home

2 Arnold M, Sierra MS, Laversanne M, *et al.* Global patterns and trends in colorectal cancer incidence and mortality. *Gut* 2017;**66**:683–91. doi:10.1136/gutjnl-2015-310912

3 Sierra MS, Forman D. Burden of colorectal cancer in Central and South America. *Cancer Epidemiol* 2016;**44**:S74–81. doi:10.1016/j.canep.2016.03.010

4 Laiyemo AO, Brawley O, Irabor D, *et al.* Towards colorectal cancer control in Africa HHS Public Access. *Int J Cancer* 2016;**138**:1033–4. doi:10.1002/ijc.29843

5 Lauby-Secretan B, Vilahur N, Bianchini F, *et al.* The IARC Perspective on Colorectal Cancer Screening. *N Engl J Med* 2018;**378**:1734–40.

6 Hawkes N. Cancer survival data emphasise importance of early diagnosis. *BMJ* 2019;**364**:l408. doi:10.1136/bmj.l408

7 Sivaram S, Majumdar G, Perin D, *et al.* Population-based cancer screening programmes in low-income and middle-income countries: regional consultation of the International Cancer Screening Network in India. *Lancet Oncol* 2018;**19**:e113–22. doi:10.1016/S1470-2045(18)30003-2

8 Schreuders EH, Ruco A, Rabeneck L, *et al.* Colorectal cancer screening: A global overview of existing programmes. *Gut* 2015;**64**:1637–49. doi:10.1136/gutjnl-2014-309086

9 Moore MA, Ab Manan A, Chow KY, *et al.* Cancer Epidemiology and Control in Peninsular and Island South-East Asia - Past, Present and Future. *Asian Pacific J Cancer Prev* 2010;**11**:81–97.

10 Altobelli E, D'Aloisio F, Angeletti PM. Colorectal cancer screening in countries of European Council outside of the EU-28. *World J Gastroenterol* 2016;**22**:4946–57.

For peer review only

For peer review only - http://bmjopen.bmj.com/site/about/guidelines.xhtml

doi:10.3748/wjg.v22.i20.4946

11 Morhason-Bello IO, Odedina F, Rebbeck TR, *et al.* Challenges and opportunities in cancer control in Africa: A perspective from the African Organisation for Research and Training in Cancer. *Lancet Oncol* 2013;**14**:e142–51. doi:10.1016/S1470-2045(12)70482-5

12 Priaulx J, Turnbull E, Heijnsdijk E, *et al.* The influence of health systems on breast, cervical and colorectal cancer screening: an overview of systematic reviews using health systems and implementation research frameworks. *J Heal Serv Res Policy* 2019;:15–7. doi:10.1177/1355819619842314

13 Davis MM, Freeman M, Shannon J, *et al.* A systematic review of clinic and community intervention to increase fecal testing for colorectal cancer in rural and low-income populations in the united states – How, what and when? *BMC Cancer* 2018;**18**:1–16. doi:10.1186/s12885-017-3813-4

14 Tricco AC, Lillie E, Zarin W, *et al.* PRISMA extension for scoping reviews (PRISMA-ScR): Checklist and explanation. *Ann Intern Med* 2018;**169**:467–73. doi:10.7326/M18-0850

15 Arksey H, O'Malley L. Scoping studies: Towards a methodological framework. *Int J Soc Res Methodol Theory Pract* 2005;**8**:19–32. doi:10.1080/1364557032000119616

16 Hoffmann TC, Glasziou PP, Barbour V, *et al.* Better reporting of interventions : template for intervention description and replication (TIDieR) checklist and guide. *BMJ* 2014;**1687**:1–12. doi:10.1136/bmj.g1687

17 A. Tierney A, C. Haverfield M, P. McGovern M, *et al.* Advancing Evidence Synthesis from Effectiveness to Implementation: Integration of Implementation Measures into Evidence Reviews. *J Gen Intern Med* Published Online First: 2019. doi:10.1007/s11606-019-05586-3

For peer review only - http://bmjopen.bmj.com/site/about/guidelines.xhtml

Table 1 Data charting domains and explanations adapted from the TIdieR checklist [16]

**Data charting domain**	**Explanation**[a]
Reference information	Extract first author and year of publication
Brief name	Name or a phrase that describes the intervention
Why	Describe any rationale, theory, or goal of the elements essential to the intervention
What	**Study design:** e.g. cross-sectional, quasi-experimental, observational<br>**Materials:** describe any physical or informational materials used in the intervention, including those provided to participants or used in intervention delivery or in training of intervention providers. Provide information on where the materials can be accessed (such as online appendix, URL)<br>**Procedures:** describe each of the procedures, activities, and/or processes used in the intervention, including any enabling or support activities
Who provided	For each category of intervention provider (such as psychologist, nursing assistant), describe their expertise, background, and any specific training given
How	Describe the modes of delivery (such as face to face or by some other mechanism, such as internet or telephone) of the intervention and whether it was provided individually or in a group
Where	Describe the type(s) of location(s) where the intervention occurred, including any necessary infrastructure or relevant features
For whom	Describe the target audience as well as inclusion and exclusion criteria if provided. Provide information about the sampling procedure.
When and how much	Describe the number of times the intervention was delivered and over what period of time including the number of sessions, their schedule, and their duration, intensity, or dose
Tailoring	If the intervention was planned to be personalised, titrated or adapted, then describe what, why, when, and how
Modifications	If the intervention was modified during the course of the study, describe the changes (what, why, when, and how)

[a] Data may not be provided in each study. All information provided that is listed here will be extracted.

For peer review only

Table 2 Data charting domains and explanations from implementation science frameworks [17]

Data charting domain	Explanation
Acceptability	Perception among implementation stakeholders that the intervention is agreeable, palatable, or satisfactory
Adoption	Intention, initial decision, or action to try or employ an innovation or evidence-based practice
Appropriateness	Perceived fit, relevance, or compatibility of the intervention for a given practice setting, provider, target population, or problem
Feasibility	Extent to which the intervention can be successfully used or carried out within a given setting
Fidelity	Degree to which an intervention was implemented as it was prescribed in the original protocol or as it was intended (planned and actual fidelity)
Implementation cost	Cost impact of an implementation effort (influenced by intervention complexity, implementation strategy, and setting)
Intervention complexity	Perceived difficulty of implementation, reflected by duration, scope, radicalness, disruptiveness, centrality, and intricacy and number of steps required to implement
Penetration	Integration of a practice within a service setting and its subsystems
Reach	The absolute number, proportion, and representativeness of individuals who are willing to participate in an intervention
Sustainability	Extent to which a newly implemented intervention is maintained or institutionalized within a service setting's ongoing, stable operations

For peer review only

For peer review only - http://bmjopen.bmj.com/site/about/guidelines.xhtml

Supplementary material 1 : Definition of income groupings

Definition of income groupings has been sourced from the World Bank and is based on gross national income (GNI): https://datahelpdesk.worldbank.org/knowledgebase/articles/906519-world-bank-country-and-lending-groups

Low-income: Low-income economies are defined as those with a GNI per capita, calculated using the World Bank Atlas method, of $1,025 or less in 2018.

Lower-middle income: Lower-middle income economies are defined as those with a GNI per capita, calculated using the World Bank Atlas method, between $1,026 and $3,995.

Upper-middle income: Upper-middle income economies are defined as those with a GNI per capita, calculated using the World Bank Atlas method, between $3,996 and $12,375.

High-income: High-income economies are defined as those with a GNI per capita, calculated using the World Bank Atlas method, of $12,376 or more.

For peer review only

For peer review only - http://bmjopen.bmj.com/site/about/guidelines.xhtml

Supplementary materials 2 : Search terms and strategy as devised for MEDLINE

1 exp Colorectal Neoplasms/

2 bowel cancer.mp.

3 early diagnosis/ OR "early detection of cancer"/

4 Mass Screening/

5 (f$cal occult blood test OR fobt OR f$cal immunochemical test OR fit OR colonoscopy OR sigmoidoscopy).mp. [mp=title, abstract, original title, name of substance word, subject heading word, floating sub-heading word, keyword heading word, organism supplementary concept word, protocol supplementary concept word, rare disease supplementary concept word, unique identifier, synonyms]

6 Developing Countries/

7 (Angola* OR India* OR Papua New Guinea OR Bangladesh* OR Indonesia* OR Philippin* OR Bhutan OR Kenya* OR Sao Tom* OR Bolivia* OR Kiribati OR Senegal* OR Cabo Verde* OR Kyrgyz* OR Solomon Island* OR Cambodi* OR Lao* OR Sudan* OR Cameroon* OR Lesotho OR Timor Leste* OR Comoros OR Mauritania* OR Tunisia* OR Congo OR Micronesia OR Ukrain* OR Cote d'Ivoire OR Moldov* OR Uzbekistan* OR Djibouti OR Mongolia* OR Vanuatu Egypt* OR Morocc* OR Vietnam* OR El Salvador OR Myanmar* OR Gaza* OR Eswatini OR Nicaragua* OR Zambia* OR Ghana* OR Nigeria* OR Zimbabwe* OR Honduras* OR Pakistan* OR Argentin* OR Paraguay* OR Mexic* OR Venezuel* OR Costa Rica* OR Cuba* OR Jamaica* OR Guatemala* OR Peru* OR Dominican Republic* OR Ecuador* OR Colombia* OR Brazil* OR Chin* OR Sri Lanka* OR Malay* OR Thai* OR Turk* OR Iran* OR Albania* OR South Africa* OR Maurit* OR Namib* OR Algeria* OR Botswana* OR Bulgaria* OR Fiji* OR Gabon* OR Nauru* OR American Samoa* OR Georgia* OR North Macedonia* OR Grenada* OR Armenia* OR Azerbaijan* OR Guyana* OR Romania* OR Belarus* OR Russia* OR Belize* OR Iraq* OR Samoa* OR Bosnia* OR Herzegovina* OR Serbia* OR Jordan* OR Kazakhstan* OR Kosovo* OR St Lucia* OR Lebanon* OR Grenadin* OR Suriname OR Maldives OR Tonga* OR Dominica OR Marshall Islands OR Turkmenistan OR Equatorial Guinea OR Tuvalu OR Montenegro* OR Liby* OR Afghan* OR Guinea* OR Sierra Leone OR Benin* OR Haiti* OR Somalia* OR Burkina Faso* OR Korea OR South Sudan* OR Burundi* OR Liberia* OR Syria* OR Central African Republic OR Madagascar* OR Tajikistan* OR Chad* OR Malawi* OR Tanzania* OR Congo* OR Mali* OR Togo* OR Eritrea* OR Mozambique* OR Uganda* OR Ethiopia* OR Nepal OR Yemen* OR Gambia* OR Guinea* OR Rwanda*).mp. [mp=title, abstract, original title, name of substance word, subject heading word, floating sub-heading word, keyword heading word, organism supplementary concept word, protocol supplementary concept word, rare disease supplementary concept word, unique identifier, synonyms]

For peer review only - http://bmjopen.bmj.com/site/about/guidelines.xhtml

8 1 OR 2

9 3 OR 4 OR 5

10 6 OR 7

11 8 and 9 and 10

12 limit 11 to (English language and humans)

For peer review only

For peer review only - http://bmjopen.bmj.com/site/about/guidelines.xhtml

Preferred Reporting Items for Systematic reviews and Meta-Analyses extension for Scoping Reviews (PRISMA-ScR) Checklist

SECTION	ITEM	PRISMA-ScR CHECKLIST ITEM	REPORTED ON PAGE #
**TITLE**			
Title	1	Identify the report as a scoping review.	1
**ABSTRACT**			
Structured summary	2	Provide a structured summary that includes (as applicable): background, objectives, eligibility criteria, sources of evidence, charting methods, results, and conclusions that relate to the review questions and objectives.	2
**INTRODUCTION**			
Rationale	3	Describe the rationale for the review in the context of what is already known. Explain why the review questions/objectives lend themselves to a scoping review approach.	4
Objectives	4	Provide an explicit statement of the questions and objectives being addressed with reference to their key elements (e.g., population or participants, concepts, and context) or other relevant key elements used to conceptualize the review questions and/or objectives.	5
**METHODS**			
Protocol and registration	5	Indicate whether a review protocol exists; state if and where it can be accessed (e.g., a Web address); and if available, provide registration information, including the registration number.	This is the protocol manuscript.
Eligibility criteria	6	Specify characteristics of the sources of evidence used as eligibility criteria (e.g., years considered, language, and publication status), and provide a rationale.	6-7
Information sources*	7	Describe all information sources in the search (e.g., databases with dates of coverage and contact with authors to identify additional sources), as well as the date the most recent search was executed.	6
Search	8	Present the full electronic search strategy for at least 1 database, including any limits used, such that it could be repeated.	Supplementary material 2
Selection of sources of evidence†	9	State the process for selecting sources of evidence (i.e., screening and eligibility) included in the scoping review.	7
Data charting process‡	10	Describe the methods of charting data from the included sources of evidence (e.g., calibrated forms or forms that have been tested by the team before their use, and whether data charting was done independently or in duplicate) and any processes for obtaining and confirming data from investigators.	7,8
Data items	11	List and define all variables for which data were sought and any assumptions and simplifications made.	8,12,13
Critical appraisal of individual	12	If done, provide a rationale for conducting a critical appraisal of included sources of evidence; describe	n/a

For peer review only - http://bmjopen.bmj.com/site/about/guidelines.xhtml

SECTION	ITEM	PRISMA-ScR CHECKLIST ITEM	REPORTED ON PAGE #
sources of evidence§		the methods used and how this information was used in any data synthesis (if appropriate).	
Synthesis of results	13	Describe the methods of handling and summarizing the data that were charted.	8
**RESULTS**			
Selection of sources of evidence	14	Give numbers of sources of evidence screened, assessed for eligibility, and included in the review, with reasons for exclusions at each stage, ideally using a flow diagram.	n/a
Characteristics of sources of evidence	15	For each source of evidence, present characteristics for which data were charted and provide the citations.	n/a
Critical appraisal within sources of evidence	16	If done, present data on critical appraisal of included sources of evidence (see item 12).	n/a
Results of individual sources of evidence	17	For each included source of evidence, present the relevant data that were charted that relate to the review questions and objectives.	n/a
Synthesis of results	18	Summarize and/or present the charting results as they relate to the review questions and objectives.	n/a
**DISCUSSION**			
Summary of evidence	19	Summarize the main results (including an overview of concepts, themes, and types of evidence available), link to the review questions and objectives, and consider the relevance to key groups.	n/a
Limitations	20	Discuss the limitations of the scoping review process.	n/a
Conclusions	21	Provide a general interpretation of the results with respect to the review questions and objectives, as well as potential implications and/or next steps.	n/a
**FUNDING**			
Funding	22	Describe sources of funding for the included sources of evidence, as well as sources of funding for the scoping review. Describe the role of the funders of the scoping review.	9

JBI = Joanna Briggs Institute; PRISMA-ScR = Preferred Reporting Items for Systematic reviews and Meta-Analyses extension for Scoping Reviews.
* Where *sources of evidence* (see second footnote) are compiled from, such as bibliographic databases, social media platforms, and Web sites.
† A more inclusive/heterogeneous term used to account for the different types of evidence or data sources (e.g., quantitative and/or qualitative research, expert opinion, and policy documents) that may be eligible in a scoping review as opposed to only studies. This is not to be confused with *information sources* (see first footnote).
‡ The frameworks by Arksey and O'Malley (6) and Levac and colleagues (7) and the JBI guidance (4, 5) refer to the process of data extraction in a scoping review as data charting.
§ The process of systematically examining research evidence to assess its validity, results, and relevance before using it to inform a decision. This term is used for items 12 and 19 instead of "risk of bias" (which is more applicable to systematic reviews of interventions) to include and acknowledge the various sources of evidence that may be used in a scoping review (e.g., quantitative and/or qualitative research, expert opinion, and policy document).

From: Tricco AC, Lillie E, Zarin W, O'Brien KK, Colquhoun H, Levac D, et al. PRISMA Extension for Scoping Reviews (PRISMAScR): Checklist and Explanation. Ann Intern Med. 2018;169:467–473. doi: 10.7326/M18-0850.

For peer review only - http://bmjopen.bmj.com/site/about/guidelines.xhtml

BMJ Open

Implementation of colorectal cancer screening interventions in low- and middle-income countries: a scoping review protocol

Journal:	*BMJ Open*
Manuscript ID	bmjopen-2020-037520.R1
Article Type:	Protocol
Date Submitted by the Author:	06-May-2020
Complete List of Authors:	Schliemann, Desiree ; Queen's University Belfast, Centre for Public Health<br>Matovu, Nicholas ; Queen's University Belfast Centre for Public Health<br>Ramanathan, Kogila; Monash University - Malaysia Campus<br>Muñoz, Paloma; Instituto Nacional de Salud Publica, Centre for Research and Population Health<br>O'Neill, Ciaran; Queen's University Belfast, Centre for Public Health<br>Kee, Frank; Queen's University Belfast, Centre for Public Health<br>Su, Tin ; Monash University - Malaysia Campus<br>Donnelly, Michael; Queen's University Belfast, Centre for Public Health
**Primary Subject Heading**:	Global health
Secondary Subject Heading:	Oncology, Public health
Keywords:	Adult oncology < ONCOLOGY, PUBLIC HEALTH, International health services < HEALTH SERVICES ADMINISTRATION & MANAGEMENT, PREVENTIVE MEDICINE

SCHOLARONE™
Manuscripts

I, the Submitting Author has the right to grant and does grant on behalf of all authors of the Work (as defined in the below author licence), an exclusive licence and/or a non-exclusive licence for contributions from authors who are: i) UK Crown employees; ii) where BMJ has agreed a CC-BY licence shall apply, and/or iii) in accordance with the terms applicable for US Federal Government officers or employees acting as part of their official duties; on a worldwide, perpetual, irrevocable, royalty-free basis to BMJ Publishing Group Ltd ("BMJ") its licensees and where the relevant Journal is co-owned by BMJ to the co-owners of the Journal, to publish the Work in this journal and any other BMJ products and to exploit all rights, as set out in our licence.

The Submitting Author accepts and understands that any supply made under these terms is made by BMJ to the Submitting Author unless you are acting as an employee on behalf of your employer or a postgraduate student of an affiliated institution which is paying any applicable article publishing charge ("APC") for Open Access articles. Where the Submitting Author wishes to make the Work available on an Open Access basis (and intends to pay the relevant APC), the terms of reuse of such Open Access shall be governed by a Creative Commons licence – details of these licences and which Creative Commons licence will apply to this Work are set out in our licence referred to above.

Other than as permitted in any relevant BMJ Author's Self Archiving Policies, I confirm this Work has not been accepted for publication elsewhere, is not being considered for publication elsewhere and does not duplicate material already published. I confirm all authors consent to publication of this Work and authorise the granting of this licence.

For peer review only

Title: Implementation of colorectal cancer screening interventions in low- and middle-income countries: a scoping review protocol

Authors: Désirée Schliemann[1], Nicholas Matovu[1], Kogila Ramanathan[2], Paloma Muñoz-Aguirre[3], Ciaran O'Neill[2], Frank Kee[2], Tin Tin Su[2], Michael Donnelly[1]

[1] Centre for Public Health and UKCRC Centre of Excellence for Public Health, Queen's University Belfast, Belfast, UK

[2] South East Asia Community Observatory (SEACO), Jeffrey Cheah School of Medicine and Health Sciences, Monash University Malaysia

[3] Centre for Research on Population Health (CONACYT), Instituto Nacional de Salud Pública (INSP), Cuernavaca, Mexico

Corresponding author: Désirée Schliemann/ d.schliemann@qub.ac.uk

For peer review only

For peer review only - http://bmjopen.bmj.com/site/about/guidelines.xhtml

Abstract

Introduction: Colorectal cancer (CRC) imposes a significant global burden of disease. CRC survival rates are much lower in low- and middle-income countries (LMICs). Screening tends to lead to an improvement in cancer detection and the uptake of available treatments and, in turn, to better chances of cancer survival. Most evidence on CRC screening interventions comes from high-income countries. The objective of this scoping review is to map the available literature on the implementation of CRC screening interventions in LMICs.

Methods and analysis: We will conduct a scoping review according to the framework proposed by Arksey and O'Malley (2005). We will search MEDLINE, EMBASE, Web of Science and Google Scholar using a combination of terms such as 'colorectal cancer', 'screening' and 'low-middle-income countries'. Studies of CRC screening interventions/programmes conducted in the general adult population in LMICs as well as policy reviews (of interventions in LMICs) and commentaries on challenges and opportunities of delivering CRC screening in LMICs, published in the English language before February 2020 will be included in this review. The title and abstract screen will be conducted by one reviewer and two reviewers will screen full-texts and extract data from included papers, independently, into a data charting template that will include criteria from an adapted TIDieR checklist and implementation considerations. The presentation of the scoping review will be reported according to PRISMA-ScR guidance.

Ethics and dissemination: There are no ethical concerns. The results will be used to inform colorectal screening interventions in low-middle-income countries. We will publish the findings in a peer-reviewed journal and present them at relevant conferences.

Key words: colorectal cancer, bowel cancer, screening, faecal occult blood test, faecal immunochemical test, low-middle income countries

For peer review only

Strengths and limitations of this study

- This scoping review will map the literature on colorectal cancer screening in low- and middle-income countries, thus highlighting opportunities and challenges for those countries and informing future global health research.
- The approach presented here will provide a timely scoping review synthesis of the literature in this field.
- The inclusion criteria are broad in order to 'paint' a comprehensive picture.
- We will include only studies published in the English language, which may mean that programmes and interventions published in other languages will be missed.
- The review uses the World Bank classification rather than the Human Development Index to define middle-income countries - some countries that the HDI define as high-income countries may be classed as upper-middle income by the World Bank.

For peer review only

For peer review only - http://bmjopen.bmj.com/site/about/guidelines.xhtml

Introduction

Colorectal cancer (CRC) is the fourth most common cancer worldwide with an age-standardised incidence rate of 19.7/100,000 for males and females combined and it remains the third most common cause of cancer deaths globally with an estimated age-standardized mortality rate of 8.9% in 2018 [1]. Large disparities exist in CRC incidence and mortality rates between high- and low-income countries. In Oceania, Europe and North America, CRC mortality rates are about one quarter of incidence rates, whereas in Asia, Latin America and the Caribbean mortality rates represent over half of incidence rates, and in Africa mortality rates are over two-thirds of incidence rates [1]. CRC incidence and mortality correlate with the human development index (HDI) - low- and middle-income countries (LMICs) have experienced a rapid increase in CRC incidence and mortality whereas rates in high-income countries are stabilising or decreasing [2]. Rapid yearly increases in CRC incidence of up to 2% have been recorded for some middle-income countries such as Brazil and Costa Rica since the early 2000s [3] and in mortality rates in the Philippines (5.7%) and Belarus (3.7%) over a 10-year period [2]. It has also been estimated that CRC incidence in Africa is expected to increase by 85% by 2030 [4]. Arguably, aging populations and lifestyle changes that more commonly resemble Western lifestyles in LMIC are the main reasons for the increasing trends as well as advancements in cancer detection [2].

The International Agency for Cancer Research (IARC) suggest that there is sufficient evidence in relation to reduced CRC mortality to recommend screening (biannually) for CRC [5]. Screening is most effective when it is delivered in a way that detects cancer at an early stage, i.e. stage 1 or 2 [6], when CRC patients are often asymptomatic. The guaiac faecal occult blood test (gFOBT) and the faecal immunochemical test for haemoglobin (FIT) are non-invasive, inexpensive stool tests that screen for small amounts of blood and that tend to be offered to asymptomatic, ‘at-risk’ individuals (e.g. people at a certain age or with a CRC family history). Invasive, visual screening techniques such as colonoscopy and flexible sigmoidoscopy are offered, generally, to symptomatic and high-risk individuals or those with a positive gFOBT/ FIT results. In the last ten years, many high-income countries have introduced organised, population-based cancer screening programmes and follow a systematic approach whereby anyone over a certain age (e.g. 50 years) is invited to complete a stool

For peer review only - http://bmjopen.bmj.com/site/about/guidelines.xhtml

test at regular (e.g. two-yearly) intervals. Screening interventions are recommended only if high quality treatment and follow-up can be delivered [5] and, therefore, the readiness of a country's health system to implement a screening programme is crucial [7]. Some middle-income countries offer opportunistic screening, (e.g. sporadic stool tests to individuals at-risk who attend a clinic) but lack financial resources and the infrastructure to support organised screening (i.e. qualified staff, screening centres, and cancer registries). Low income countries appear to have no or limited CRC screening or treatment facilities in place [8]. Many LMICs also lack cancer registries and, therefore, recording of cases and cancer control efforts are a major challenge [9–11]. Thus, evaluation reports of CRC screening interventions come mainly from high-income countries and most CRC interventions and programmes were delivered at least partly through post, i.e. invitation letters to attend screening and/or mailed stool-test kits to be completed at home [12,13]. The discrepancies between CRC incidence globally and the availability of opportunistic or population-based screening remain a concern [8] and the rising CRC incidence and high mortality rates in LMICs countries require efforts to improve CRC outcomes.

Review objectives

The purpose of this scoping review is to review the global health literature, map evidence from peer-reviewed and grey literature, examine programmes and interventions that aim to improve CRC screening uptake in LMICs countries and highlight research gaps and opportunities in order to answer the three review questions below. Findings will be used to guide future CRC screening efforts in countries with limited resources. We will use the classification by the World Bank to define countries in terms of lower-middle income, upper-middle income and low-income economies (Supplementary materials 1).

Methods and analysis

We will follow the research approach that conforms with the Preferred Reporting Items for Systematic Reviews and Meta-Analysis extension for Scoping Reviews (PRISMA-ScR) [14]. A scoping review is

For peer review only - http://bmjopen.bmj.com/site/about/guidelines.xhtml

the most appropriate type of review given the underexplored nature of this area and the need for iterative-style searching of multiple sources. The scoping review will be guided by the methodological five-step framework developed by Arksey and O'Malley [15] as outlined below.

Stage 1: identifying the research question

The review will aim to scope literature in the public domain regarding I) the content delivered, implementation and uptake of CRC screening interventions and programmes in LMICs and II) challenges and opportunities for CRC screening in LMICs. In particular, the review will attempt to answer the following research questions:

1) What interventions/programmes have been implemented in LMICs that were designed explicitly to encourage people to attend CRC screening (including interventions implemented at a patient-, provider- and system-level)?
2) What are the opportunities and challenges in terms of implementing CRC screening interventions/ programmes in LMICs?
3) What are the findings (qualitative and quantitative outcomes) from interventions/programmes in terms of, for example, implementation, reach, uptake, engagement (including differences between rural and urban areas) and costs/resources?

A scoping rather than a systematic review was the chosen methodological approach as this topic is unexplored and, so, the main aim of the review is to scope the landscape of relevant reports and to address the above noted broad questions as well as identify whether or not there is a need, or there are sufficient studies, to conduct a systematic review of the effectiveness or cost-effectiveness of CRC screening interventions.

For peer review only - http://bmjopen.bmj.com/site/about/guidelines.xhtml

Stage 2: identifying relevant studies

We will search MEDLINE, EMBASE, Web of Science, and Google Scholar for relevant literature. Furthermore, we will hand search the reference lists of relevant reviews and studies and Google Web for unpublished reports and briefings. In addition, we will contact experts in the field of CRC screening in LMIC to identify additional programmes that may not have been published in the scientific literature. We have devised an initial search strategy (with the expertise of an experienced subject librarian) for MEDLINE (Supplementary material 2) which will be adapted for other databases. Search terms revolve around the three categories related to 'colorectal cancer', 'screening' and 'LMIC countries.

Stage 3: study selection

The lead author (DS) will conduct the title and abstract screen and two authors (DS and NM or PM) will separately screen the retrieved full-texts for inclusion in this scoping review based on broad inclusion and exclusion criteria. Inconsistencies between reviewers will be discussed with a third reviewer (MD).

This scoping review will focus on two types of literature; I) dedicated empirical studies of CRC screening interventions or programmes that were explicitly designed to encourage the public to attend CRC screening and II) commentaries/editorials and policy reviews that discuss opportunities and challenges for CRC screening implementation in LMICs. National screening programmes as well as screening programmes and interventions delivered at lower population aggregates and programmes of any duration published before February 2020 will be included. Only studies conducted in LMICs targeting the general adult population (18 years or older) or asymptomatic populations 'at-risk' for CRC will be considered for inclusion (this may vary between countries, but is often referred to as adults aged over 40 or 50 years). Studies aiming to improve cancer screening amongst cancer patients and health care professionals will be excluded from this review given their advanced understanding and direct experience of the importance of cancer screening compared to the general population. However, interventions targeted at health care professionals with the aim of improving screening amongst the

For peer review only - http://bmjopen.bmj.com/site/about/guidelines.xhtml

general population will be considered for inclusion. We will exclude protocols if no further information on the implementation of the programme is available. We will only include studies published in the English language and will not apply restrictions concerning the year of publication.

Stage 4: charting the data

We will chart the data from the included intervention studies/programme in a descriptive manner according to categories in an adapted Template for Intervention Description and Replication (TIDieR) checklist for the reporting of interventions [16] in order to illuminate key components and facilitate the production of standardised descriptions. The following fields will be added to the domains of the TIDieR checklist and charted accordingly: 'reference information', 'study design' and 'for whom' (i.e. target population) (Table 1). We will also chart data descriptively on implementation considerations to identify key concepts that influence dissemination and successful implementation of evidence-based programmes. This will be guided by a template developed by Tierney et al [17] which includes ten factors (Table 2), one of which overlaps with the TIDieR checklist (i.e. intervention 'fidelity') and which therefore will only be addressed under implementation considerations. Both checklists have been piloted and deemed appropriate by the team. Two authors (DS and NM, KR or PM) will independently extract information and any discrepancies will be resolved in discussion with a third author (MD or TTS). In addition, themes will be extracted qualitatively from reviews and commentaries/editorials about the implementation challenges and opportunities of CRC screening programmes in LMICs.

Stage 5: collating, summarising and reporting the results

We will summarise the data extracted from each study and present the findings in a series of detailed tables that will be organised according the TIDieR and implementation science domains, and marshalled to the content of each review question (e.g. intervention) and then grouped or aggregated according to country, region and income level. In particular, the review will present the barriers and enablers to the successful implementation and uptake of CRC screening in LMICs and resource-constrained settings.

For peer review only - http://bmjopen.bmj.com/site/about/guidelines.xhtml

Patient and public involvement

The results of the protocol will be shared and discussed with key stakeholders in various global health studies, particularly in relation to our studies in rural communities in Malaysia (i.e. the Ministry of Health and non-governmental cancer organisations that provide CRC screening services).

Ethics and dissemination

This paper presents the protocol for a scoping review of CRC screening interventions in low- and middle-income countries. Ethics approval is not necessary as the data will be collected from publically available resources. This review will advance the knowledge on CRC screening programmes for researchers, general practitioners, public health practitioners and policy makers working in low- and middle-income countries. The results will be disseminated through a peer-reviewed journal and presented at relevant conferences. Furthermore, this scoping review will be conducted to inform the implementation of a CRC pilot screening intervention for Malaysia.

For peer review only

For peer review only - http://bmjopen.bmj.com/site/about/guidelines.xhtml

Contributors

DS, MD and TTS conceived the idea for the scoping review. DS and MD developed the research question. DS, NM, KR, PM, CO, FK, TTS and MD aided in developing the scoping review methods. DS drafted the manuscript and MD led the editing of the manuscript. All authors revised the manuscript critically for intellectual content and gave final approval of the submitted and revised manuscript.

Funding

This research is funded by the Medical Research Council (UK) Global Challenges Research Fund.

Competing interests

None declared.

Patient consent for publication

Not required.

For peer review only

For peer review only - http://bmjopen.bmj.com/site/about/guidelines.xhtml

References

1 International Agency for Research on Cancer. GLOBOCAN. 2018. http://gco.iarc.fr/today/home

2 Arnold M, Sierra MS, Laversanne M, *et al.* Global patterns and trends in colorectal cancer incidence and mortality. *Gut* 2017;**66**:683–91. doi:10.1136/gutjnl-2015-310912

3 Sierra MS, Forman D. Burden of colorectal cancer in Central and South America. *Cancer Epidemiol* 2016;**44**:S74–81. doi:10.1016/j.canep.2016.03.010

4 Laiyemo AO, Brawley O, Irabor D, *et al.* Towards colorectal cancer control in Africa HHS Public Access. *Int J Cancer* 2016;**138**:1033–4. doi:10.1002/ijc.29843

5 Lauby-Secretan B, Vilahur N, Bianchini F, *et al.* The IARC Perspective on Colorectal Cancer Screening. *N Engl J Med* 2018;**378**:1734–40.

6 Hawkes N. Cancer survival data emphasise importance of early diagnosis. *BMJ* 2019;**364**:l408. doi:10.1136/bmj.l408

7 Sivaram S, Majumdar G, Perin D, *et al.* Population-based cancer screening programmes in low-income and middle-income countries: regional consultation of the International Cancer Screening Network in India. *Lancet Oncol* 2018;**19**:e113–22. doi:10.1016/S1470-2045(18)30003-2

8 Schreuders EH, Ruco A, Rabeneck L, *et al.* Colorectal cancer screening: A global overview of existing programmes. *Gut* 2015;**64**:1637–49. doi:10.1136/gutjnl-2014-309086

9 Moore MA, Ab Manan A, Chow KY, *et al.* Cancer Epidemiology and Control in Peninsular and Island South-East Asia - Past, Present and Future. *Asian Pacific J Cancer Prev* 2010;**11**:81–97.

10 Altobelli E, D'Aloisio F, Angeletti PM. Colorectal cancer screening in countries of European Council outside of the EU-28. *World J Gastroenterol* 2016;**22**:4946–57.

For peer review only

For peer review only - http://bmjopen.bmj.com/site/about/guidelines.xhtml

doi:10.3748/wjg.v22.i20.4946

11 Morhason-Bello IO, Odedina F, Rebbeck TR, *et al.* Challenges and opportunities in cancer control in Africa: A perspective from the African Organisation for Research and Training in Cancer. *Lancet Oncol* 2013;**14**:e142–51. doi:10.1016/S1470-2045(12)70482-5

12 Priaulx J, Turnbull E, Heijnsdijk E, *et al.* The influence of health systems on breast, cervical and colorectal cancer screening: an overview of systematic reviews using health systems and implementation research frameworks. *J Heal Serv Res Policy* 2019;:15–7. doi:10.1177/1355819619842314

13 Davis MM, Freeman M, Shannon J, *et al.* A systematic review of clinic and community intervention to increase fecal testing for colorectal cancer in rural and low-income populations in the united states – How, what and when? *BMC Cancer* 2018;**18**:1–16. doi:10.1186/s12885-017-3813-4

14 Tricco AC, Lillie E, Zarin W, *et al.* PRISMA extension for scoping reviews (PRISMA-ScR): Checklist and explanation. *Ann Intern Med* 2018;**169**:467–73. doi:10.7326/M18-0850

15 Arksey H, O'Malley L. Scoping studies: Towards a methodological framework. *Int J Soc Res Methodol Theory Pract* 2005;**8**:19–32. doi:10.1080/1364557032000119616

16 Hoffmann TC, Glasziou PP, Barbour V, *et al.* Better reporting of interventions : template for intervention description and replication (TIDieR) checklist and guide. *BMJ* 2014;**1687**:1–12. doi:10.1136/bmj.g1687

17 A. Tierney A, C. Haverfield M, P. McGovern M, *et al.* Advancing Evidence Synthesis from Effectiveness to Implementation: Integration of Implementation Measures into Evidence Reviews. *J Gen Intern Med* Published Online First: 2019. doi:10.1007/s11606-019-05586-3

For peer review only - http://bmjopen.bmj.com/site/about/guidelines.xhtml

Table 1 Data charting domains and explanations adapted from the TIdieR checklist

**Data charting domain**	**Explanation**[a]
Reference information	Extract first author and year of publication
Brief name	Name or a phrase that describes the intervention
Why	Describe any rationale, theory, or goal of the elements essential to the intervention
What	**Study design:** e.g. cross-sectional, quasi-experimental, observational<br>**Materials:** describe any physical or informational materials used in the intervention, including those provided to participants or used in intervention delivery or in training of intervention providers. Provide information on where the materials can be accessed (such as online appendix, URL)<br>**Procedures:** describe each of the procedures, activities, and/or processes used in the intervention, including any enabling or support activities
Who provided	For each category of intervention provider (such as psychologist, nursing assistant), describe their expertise, background, and any specific training given
How	Describe the modes of delivery (such as face to face or by some other mechanism, such as internet or telephone) of the intervention and whether it was provided individually or in a group
Where	Describe the type(s) of location(s) where the intervention occurred, including any necessary infrastructure or relevant features
For whom	Describe the target audience as well as inclusion and exclusion criteria if provided. Provide information about the sampling procedure.
When and how much	Describe the number of times the intervention was delivered and over what period of time including the number of sessions, their schedule, and their duration, intensity, or dose
Tailoring	If the intervention was planned to be personalised, titrated or adapted, then describe what, why, when, and how
Modifications	If the intervention was modified during the course of the study, describe the changes (what, why, when, and how)

[a] Data may not be provided in each study. All information provided that is listed here will be extracted descriptively.

For peer review only

For peer review only - http://bmjopen.bmj.com/site/about/guidelines.xhtml

Table 2 Data charting domains and explanations from implementation science frameworks

Data charting domain[a]	Explanation
Acceptability	Perception among implementation stakeholders that the intervention is agreeable, palatable, or satisfactory
Adoption	Intention, initial decision, or action to try or employ an innovation or evidence-based practice
Appropriateness	Perceived fit, relevance, or compatibility of the intervention for a given practice setting, provider, target population, or problem
Feasibility	Extent to which the intervention can be successfully used or carried out within a given setting
Fidelity	Degree to which an intervention was implemented as it was prescribed in the original protocol or as it was intended (planned and actual fidelity)
Implementation cost	Cost impact of an implementation effort (influenced by intervention complexity, implementation strategy, and setting)
Intervention complexity	Perceived difficulty of implementation, reflected by duration, scope, radicalness, disruptiveness, centrality, and intricacy and number of steps required to implement
Penetration	Integration of a practice within a service setting and its subsystems
Reach	The absolute number, proportion, and representativeness of individuals who are willing to participate in an intervention
Sustainability	Extent to which a newly implemented intervention is maintained or institutionalized within a service setting's ongoing, stable operations

[a] Data may not be provided in each study. All information provided that is listed here will be extracted descriptively.

For peer review only

Supplementary materials 1: Definition of income groupings

Definition of income groupings has been sourced from the World Bank and is based on gross national income (GNI): https://datahelpdesk.worldbank.org/knowledgebase/articles/906519-world-bank-country-and-lending-groups

Low-income: Low-income economies are defined as those with a GNI per capita, calculated using the World Bank Atlas method, of $1,025 or less in 2018.

Lower-middle income: Lower-middle income economies are defined as those with a GNI per capita, calculated using the World Bank Atlas method, between $1,026 and $3,995.

Upper-middle income: Upper-middle income economies are defined as those with a GNI per capita, calculated using the World Bank Atlas method, between $3,996 and $12,375.

High-income: High-income economies are defined as those with a GNI per capita, calculated using the World Bank Atlas method, of $12,376 or more.

For peer review only

For peer review only - http://bmjopen.bmj.com/site/about/guidelines.xhtml

Supplementary materials 2: Search terms and strategy as devised for MEDLINE

1 exp Colorectal Neoplasms/

2 bowel cancer.mp.

3 early diagnosis/ OR "early detection of cancer"/

4 Mass Screening/

5 (f$cal occult blood test OR fobt OR f$cal immunochemical test OR fit OR colonoscopy OR sigmoidoscopy).mp. [mp=title, abstract, original title, name of substance word, subject heading word, floating sub-heading word, keyword heading word, organism supplementary concept word, protocol supplementary concept word, rare disease supplementary concept word, unique identifier, synonyms]

6 Developing Countries/

7 (Angola* OR India* OR Papua New Guinea OR Bangladesh* OR Indonesia* OR Philippin* OR Bhutan OR Kenya* OR Sao Tom* OR Bolivia* OR Kiribati OR Senegal* OR Cabo Verde* OR Kyrgyz* OR Solomon Island* OR Cambodi* OR Lao* OR Sudan* OR Cameroon* OR Lesotho OR Timor Leste* OR Comoros OR Mauritania* OR Tunisia* OR Congo OR Micronesia OR Ukrain* OR Cote d'Ivoire OR Moldov* OR Uzbekistan* OR Djibouti OR Mongolia* OR Vanuatu Egypt* OR Morocc* OR Vietnam* OR El Salvador OR Myanmar* OR Gaza* OR Eswatini OR Nicaragua* OR Zambia* OR Ghana* OR Nigeria* OR Zimbabwe* OR Honduras* OR Pakistan* OR Argentin* OR Paraguay* OR Mexic* OR Venezuel* OR Costa Rica* OR Cuba* OR Jamaica* OR Guatemala* OR Peru* OR Dominican Republic* OR Ecuador* OR Colombia* OR Brazil* OR Chin* OR Sri Lanka* OR Malay* OR Thai* OR Turk* OR Iran* OR Albania* OR South Africa* OR Maurit* OR Namib* OR Algeria* OR Botswana* OR Bulgaria* OR Fiji* OR Gabon* OR Nauru* OR American Samoa* OR Georgia* OR North Macedonia* OR Grenada* OR Armenia* OR Azerbaijan* OR Guyana* OR Romania* OR Belarus* OR Russia* OR Belize* OR Iraq* OR Samoa* OR Bosnia* OR Herzegovina* OR Serbia* OR Jordan* OR Kazakhstan* OR Kosovo* OR St Lucia* OR Lebanon* OR Grenadin* OR Suriname OR Maldives OR Tonga* OR Dominica OR Marshall Islands OR Turkmenistan OR Equatorial Guinea OR Tuvalu OR Montenegro* OR Liby* OR Afghan* OR Guinea* OR Sierra Leone OR Benin* OR Haiti* OR Somalia* OR Burkina Faso* OR Korea OR South Sudan* OR Burundi* OR Liberia* OR Syria* OR Central African Republic OR Madagascar* OR Tajikistan* OR Chad* OR Malawi* OR Tanzania* OR Congo* OR Mali* OR Togo* OR Eritrea* OR Mozambique* OR Uganda* OR Ethiopia* OR Nepal OR Yemen* OR Gambia* OR Guinea* OR Rwanda*).mp. [mp=title, abstract, original title, name of substance word, subject heading word, floating sub-heading word, keyword heading word, organism supplementary concept word, protocol supplementary concept word, rare disease supplementary concept word, unique identifier, synonyms]

For peer review only - http://bmjopen.bmj.com/site/about/guidelines.xhtml

8 1 OR 2

9 3 OR 4 OR 5

10 6 OR 7

11 8 and 9 and 10

12 limit 11 to (English language and humans)

For peer review only

For peer review only - http://bmjopen.bmj.com/site/about/guidelines.xhtml

Preferred Reporting Items for Systematic reviews and Meta-Analyses extension for Scoping Reviews (PRISMA-ScR) Checklist

SECTION	ITEM	PRISMA-ScR CHECKLIST ITEM	REPORTED ON PAGE #
**TITLE**			
Title	1	Identify the report as a scoping review.	1
**ABSTRACT**			
Structured summary	2	Provide a structured summary that includes (as applicable): background, objectives, eligibility criteria, sources of evidence, charting methods, results, and conclusions that relate to the review questions and objectives.	2
**INTRODUCTION**			
Rationale	3	Describe the rationale for the review in the context of what is already known. Explain why the review questions/objectives lend themselves to a scoping review approach.	4
Objectives	4	Provide an explicit statement of the questions and objectives being addressed with reference to their key elements (e.g., population or participants, concepts, and context) or other relevant key elements used to conceptualize the review questions and/or objectives.	5
**METHODS**			
Protocol and registration	5	Indicate whether a review protocol exists; state if and where it can be accessed (e.g., a Web address); and if available, provide registration information, including the registration number.	This is the protocol manuscript.
Eligibility criteria	6	Specify characteristics of the sources of evidence used as eligibility criteria (e.g., years considered, language, and publication status), and provide a rationale.	6-7
Information sources*	7	Describe all information sources in the search (e.g., databases with dates of coverage and contact with authors to identify additional sources), as well as the date the most recent search was executed.	6
Search	8	Present the full electronic search strategy for at least 1 database, including any limits used, such that it could be repeated.	Supplementary material 2
Selection of sources of evidence†	9	State the process for selecting sources of evidence (i.e., screening and eligibility) included in the scoping review.	7
Data charting process‡	10	Describe the methods of charting data from the included sources of evidence (e.g., calibrated forms or forms that have been tested by the team before their use, and whether data charting was done independently or in duplicate) and any processes for obtaining and confirming data from investigators.	7,8
Data items	11	List and define all variables for which data were sought and any assumptions and simplifications made.	8,12,13
Critical appraisal of individual	12	If done, provide a rationale for conducting a critical appraisal of included sources of evidence; describe	n/a

SECTION	ITEM	PRISMA-ScR CHECKLIST ITEM	REPORTED ON PAGE #
sources of evidence§		the methods used and how this information was used in any data synthesis (if appropriate).	
Synthesis of results	13	Describe the methods of handling and summarizing the data that were charted.	8
**RESULTS**			
Selection of sources of evidence	14	Give numbers of sources of evidence screened, assessed for eligibility, and included in the review, with reasons for exclusions at each stage, ideally using a flow diagram.	n/a
Characteristics of sources of evidence	15	For each source of evidence, present characteristics for which data were charted and provide the citations.	n/a
Critical appraisal within sources of evidence	16	If done, present data on critical appraisal of included sources of evidence (see item 12).	n/a
Results of individual sources of evidence	17	For each included source of evidence, present the relevant data that were charted that relate to the review questions and objectives.	n/a
Synthesis of results	18	Summarize and/or present the charting results as they relate to the review questions and objectives.	n/a
**DISCUSSION**			
Summary of evidence	19	Summarize the main results (including an overview of concepts, themes, and types of evidence available), link to the review questions and objectives, and consider the relevance to key groups.	n/a
Limitations	20	Discuss the limitations of the scoping review process.	n/a
Conclusions	21	Provide a general interpretation of the results with respect to the review questions and objectives, as well as potential implications and/or next steps.	n/a
**FUNDING**			
Funding	22	Describe sources of funding for the included sources of evidence, as well as sources of funding for the scoping review. Describe the role of the funders of the scoping review.	9

JBI = Joanna Briggs Institute; PRISMA-ScR = Preferred Reporting Items for Systematic reviews and Meta-Analyses extension for Scoping Reviews.
* Where *sources of evidence* (see second footnote) are compiled from, such as bibliographic databases, social media platforms, and Web sites.
† A more inclusive/heterogeneous term used to account for the different types of evidence or data sources (e.g., quantitative and/or qualitative research, expert opinion, and policy documents) that may be eligible in a scoping review as opposed to only studies. This is not to be confused with *information sources* (see first footnote).
‡ The frameworks by Arksey and O'Malley (6) and Levac and colleagues (7) and the JBI guidance (4, 5) refer to the process of data extraction in a scoping review as data charting.
§ The process of systematically examining research evidence to assess its validity, results, and relevance before using it to inform a decision. This term is used for items 12 and 19 instead of "risk of bias" (which is more applicable to systematic reviews of interventions) to include and acknowledge the various sources of evidence that may be used in a scoping review (e.g., quantitative and/or qualitative research, expert opinion, and policy document).

From: Tricco AC, Lillie E, Zarin W, O'Brien KK, Colquhoun H, Levac D, et al. PRISMA Extension for Scoping Reviews (PRISMAScR): Checklist and Explanation. Ann Intern Med. 2018;169:467–473. doi: 10.7326/M18-0850.

For peer review only - http://bmjopen.bmj.com/site/about/guidelines.xhtml
